# Participatory Digital Twins for Chronic Care: From Predictive Models to Shared Sensemaking

**DOI:** 10.2196/92721

**Published:** 2026-06-23

**Authors:** Qingrui Li, Bo Xie, Eric L Johnson, Juan Li

**Affiliations:** 1 Computer Science Department, North Dakota State University, 1320 Albrecht Boulevard, Fargo, ND, 58105, United States, 1 7012319662; 2 School of Nursing, The University of Texas at Austin, Austin, TX, United States; 3 School of Information, The University of Texas at Austin, Austin, TX, United States; 4 Department of Family and Community Medicine, School of Medicine and Health Sciences, University of North Dakota, Grand Forks, ND, United States

**Keywords:** participatory artificial intelligence, digital twins, chronic care, shared sensemaking, human-centered artificial intelligence, human-centered AI, artificial intelligence, AI

## Abstract

Health digital twins, computational models that integrate longitudinal data, simulation, and forecasting, are increasingly proposed as tools for chronic care management. However, most current implementations are expert oriented, prioritizing technical optimization and clinical prediction while offering limited support for patient understanding, engagement, or participation. This orientation is particularly misaligned with chronic care, which unfolds largely outside clinical settings and depends on patients’ daily decisions, social context, and sustained engagement over time. In this viewpoint, we argue for reframing digital twins as participatory systems that support shared sensemaking among patients, caregivers, and clinicians rather than functioning solely as directive, expert-facing tools. We propose a conceptual framework that positions participatory digital twins as boundary objects capable of bridging computational models, clinical reasoning, and lived experience. Within this framework, generative artificial intelligence serves as a translation and interaction layer, enabling plain-language dialogue, exploration of uncertainty, and “what-if” reasoning that allows users to interpret model outputs in relation to their own contexts, goals, and constraints. We outline key design principles for participatory digital twins, including visible uncertainty, negotiated rather than prescriptive care, mechanisms for incorporating patient context and social drivers of health, and governance structures that support accountability and recourse. At the same time, this approach depends on meaningful opportunities for participation; appropriate safeguards around generative interaction; and careful attention to privacy, consent, and uneven access to digital resources. By shifting the focus from optimization alone to understanding, interaction, and trust, participatory digital twins offer a pathway toward more equitable, human-centered, and sustainable models of artificial intelligence–enabled chronic care.

## Introduction

Digital twins have rapidly emerged as a promising paradigm in health care, particularly for chronic care management [1,2]. By integrating longitudinal data, computational models, and simulation capabilities, health digital twins aim to represent an individual’s evolving physiological state and support forecasting, scenario analysis, and clinical decision-making. In managing chronic conditions such as diabetes, hypertension, and cardiovascular disease, where day-to-day self-management, longitudinal monitoring, and repeated behavioral trade-offs are central, digital twins offer the potential to shift from episodic care toward continuous, data-informed management [3-5].

However, to date, most health digital twin efforts have been designed primarily as expert-oriented systems [2,6]. Their core functionalities—prediction, simulation, and optimization—are typically intended for interpretation and use by clinicians, researchers, or health systems, with success evaluated using technical performance metrics such as accuracy or predictive error [6]. In this context, “human in the loop” most often refers to clinical oversight rather than meaningful engagement by patients or caregivers.

This orientation presents a critical limitation for chronic care. Unlike acute conditions, chronic care is continuous; unfolds largely outside of clinical settings; and depends heavily on patients’ daily decisions, contextual knowledge, and sustained engagement [7-9]. Patients are not merely data sources for digital twins; they are the individuals who live with uncertainty, interpret bodily signals, and negotiate trade-offs between medical recommendations and everyday life. When digital twins function as opaque or directive tools, they risk reinforcing passive roles for patients, undermining trust, and limiting their real-world impact.

At the same time, advances in generative artificial intelligence (GenAI), particularly large language models (LLMs), have created new opportunities to reimagine how people interact with complex computational systems [10,11]. In the context of participatory digital twins (PDTs), the value of GenAI lies less in generating de novo medical advice than in serving as a constrained translation and interaction layer around model-based outputs, helping users ask questions, surface missing context, and explore alternative scenarios in plain language. However, this role should not be treated uncritically. In health contexts, LLMs may hallucinate facts; present incorrect statements with unwarranted fluency; produce variable responses across prompts or sessions; and communicate uncertainty unreliably, particularly when asked to express risk numerically or probabilistically [11-13]. For this reason, any use of GenAI in PDTs should remain grounded in traceable model outputs, explicitly uncertainty aware, and subject to appropriate clinical oversight rather than positioned as a stand-alone source of medical advice [12,14,15].

This shift is also timely because members of the public are increasingly turning to general-purpose LLMs for health information and advice. Recent survey studies suggest that LLM-based chatbots are already being used for health queries by a substantial minority of users, particularly younger adults, whereas broader US survey data indicate that artificial intelligence (AI) chatbots have become an emerging source of health information for the public. This trend does not make general-purpose LLM use clinically reliable; rather, it underscores the urgency of developing health AI systems that offer bounded interaction, traceable outputs, explicit uncertainty, and clinician-grounded sensemaking instead of decontextualized stand-alone chatbot advice. In this respect, PDTs can be understood not simply as another application of conversational AI but as a more accountable sociotechnical response to a landscape in which conversational systems are already shaping how people seek and interpret health guidance.

This reframing should be understood as building on rather than displacing established traditions of participatory design, co-design, and patient-centered technology development in health informatics [16-19]. Prior work in these traditions has shown that patients, caregivers, and clinicians can contribute experiential, practical, and organizational knowledge that improves the relevance and usability of health technologies. In particular, participatory design has emphasized the active involvement of users in shaping technologies that affect care, whereas co-design scholarship has framed such work as a form of collective creativity and shared problem formulation [18,20]. PDTs extend this literature in a more specific direction: they are concerned not only with who participates in designing a system but also with how participation is sustained during system use, when model outputs, uncertainty, contextual annotations, and care trade-offs must be interpreted and negotiated over time [19]. In this sense, PDTs move participation from a primarily design-stage methodology toward an ongoing, model-mediated practice of shared sensemaking in chronic care.

In this viewpoint, we argue for a reframing of health digital twins as participatory systems for chronic care. We propose that digital twins should be designed not only to predict or simulate future states but also to support shared understanding; patient and caregiver agency; and collaborative decision-making among patients, caregivers, and clinicians. Drawing on principles from participatory medicine and human-centered AI, we introduce a conceptual framework for PDTs and outline design principles that prioritize interaction, transparency, and trust. Through this reframing, we aim to highlight how PDTs can contribute to more equitable, sustainable, and democratically governed chronic care.

## Limitations of Expert-Oriented Digital Twins in Chronic Care

Health digital twins have advanced rapidly in technical sophistication, integrating diverse data streams, physiological models, and simulation capabilities to support prediction and decision-making [1,2]. Despite these advances, most existing digital twin implementations in chronic care remain expert oriented by design [2,6]. Their interfaces, outputs, and evaluation frameworks primarily assume users with clinical or technical expertise, leaving limited room for meaningful patient interaction, interpretation, or participation.

A central limitation of expert-oriented digital twins lies in their reliance on opaque or directive outputs [21,22]. Predictions or recommendations are often presented as single-point alerts, optimized actions, or summarized risk scores, with minimal explanation of underlying assumptions, uncertainty, or contextual dependencies. For patients managing chronic conditions in everyday life, such outputs can be difficult to reconcile with lived experience. When system reasoning is hidden, patients may be unable to assess whether a recommendation aligns with their immediate context (eg, a skipped meal, acute stress, or unexpected physical activity) or long-term goals, leading to confusion, mistrust, or disengagement [23,24].

Expert-oriented designs also tend to treat patient involvement primarily as data contribution rather than knowledge participation or decision-making [1,6]. Patients generate large volumes of longitudinal data through wearables, sensors, and self-reports, yet their experiential insights, such as stress, fatigue, social obligations, or deviations from routine, are rarely incorporated in ways that influence model interpretation or behavior. As a result, digital twins may accurately capture physiological signals while overlooking contextual and social drivers of health—such as caregiving demands, work conditions, or social support—that shape real-world behaviors and outcomes. Consequently, a digital twin may be mathematically robust but ecologically invalid, limiting its usefulness in real-world chronic care [25].

This asymmetry has important implications for patient behavior and engagement. Effective chronic care depends not only on accurate forecasting or simulation but also on patients’ understanding of why certain trajectories are predicted and how alternative actions may influence outcomes [26]. Systems that prioritize optimization over comprehension risk reinforcing passive roles for patients, positioning them as recipients of algorithmic directives rather than active partners in care. Over time, this dynamic can erode trust, reduce adherence, and undermine sustained engagement.

Finally, expert-oriented digital twins are typically evaluated using technical performance metrics such as prediction accuracy, error reduction, or computational efficiency that fail to capture participatory value [27,28]. These metrics provide little insight into whether patients understand model outputs, feel confident using them, or perceive the system as supporting shared decision-making. Even highly accurate digital twins may have limited real-world value if patients do not understand, trust, or meaningfully use their outputs in care [28,29]. Without evaluation criteria that reflect patient engagement, patients’ ability to understand and reason about model outputs, and trust, the benefits of digital twins may remain confined to controlled clinical settings rather than extending into everyday chronic care.

Taken together, these limitations suggest that advancing digital twins for chronic care requires more than incremental improvements in modeling or simulation. It calls for a fundamental reorientation toward participatory interaction, in which patients are recognized as knowledgeable stakeholders who can interpret, question, and shape digital twin–driven insights. This shift sets the stage for redefining digital twins as shared systems rather than expert-only tools.

## Defining PDTs

To address the limitations of expert-oriented digital twins in chronic care, we propose the concept of PDTs. Rather than positioning digital twins solely as technical systems for prediction or simulation, PDTs are designed as shared sociotechnical systems that enable patients, caregivers, and clinicians to jointly explore, interpret, and reason about health trajectories over time.

Therefore, PDTs are not intended as a substitute for participatory design or co-design approaches. Rather, they build on those traditions while addressing a problem that becomes especially salient in data-intensive, model-driven chronic care: participation must continue after deployment, at the point where predictions are produced, uncertainty is encountered, and recommendations are interpreted in everyday life [16,19,20]. A system may be co-designed during development and still function as an opaque, expert-oriented artifact in use. PDTs seek to address this gap by treating participation not only as an upstream design principle but also as a downstream interpretive practice through which patients, caregivers, and clinicians can question, contextualize, and negotiate model-driven insights over time.

At the core of this reframing is a shift in how participation is understood. In most existing implementations, patient participation is implicit and largely confined to data generation (ie, patients contribute data), whereas interpretation and decision-making remain centralized within expert workflows. PDTs, in contrast, treat patients as epistemic agents: individuals capable of generating, interpreting, and applying knowledge about their own bodies in collaboration with clinical expertise. This framing recognizes that patients possess contextual and experiential knowledge—such as symptoms, stressors, routines, preferences, and social circumstances—that is essential for meaningful chronic care but is often absent from purely clinician-driven representations.

However, caregiver participation should not be assumed to be equivalent to direct patient input. Caregivers often contribute important observational, relational, and logistical knowledge about daily routines, functional changes, treatment adherence, and the social context of care. Such input can substantially enrich interpretation, particularly when chronic care is practically shared or when a patient’s ability to engage directly is temporary, fluctuating, or limited. At the same time, caregiver accounts remain second-person perspectives rather than direct expressions of the patient’s lived experience, priorities, or preferences. For this reason, PDTs should treat caregiver input as complementary, role specific, and explicitly attributed rather than interchangeable with patient-reported knowledge. Mediated participation may be appropriate when direct patient engagement is constrained by age, illness severity, cognitive or communication limitations, or other barriers to sustained interaction; however, the scope of such substitution should remain transparent and, where possible, distinguish among observed circumstances, inferred needs, and explicitly expressed patient goals. In this way, caregiver involvement can support participatory sensemaking without collapsing proxy contribution into first-person patient agency.

PDTs function as boundary objects [30] that bridge diverse forms of expertise. In this context, the term is used in a more specific sense than simply referring to a mediating artifact or communication aid. PDTs have a shared underlying structure that is a common model, data substrate, and evolving representation of the patient’s condition while still allowing for interpretive flexibility across stakeholders. Clinicians may use the twin to examine risk trajectories and treatment implications; patients and caregivers may use the same representation to understand trade-offs, surface missing context, and relate model outputs to daily life. PDTs qualify as boundary objects not merely because they sit between groups but because they enable coordination across communities of practice without requiring those communities to adopt identical interpretations, vocabularies, or priorities.

A defining feature of PDTs is their support for interactive sensemaking. Rather than delivering static predictions or optimized recommendations, PDTs enable users to ask questions, explore alternatives, and examine uncertainty. Patients can engage in “what-if” reasoning, reflect on how contextual factors influence outcomes, and negotiate trade-offs between competing goals (eg, maintaining strict glycemic control vs enjoying a holiday meal with family). Through this process, the digital twin becomes a tool for understanding and learning, not merely for compliance.

Importantly, participation in this context does not imply transferring clinical responsibility to patients or diminishing the role of professional expertise. Instead, PDTs are intended to reconfigure collaboration in chronic care. Clinicians retain their interpretive and decision-making authority, whereas patients gain greater insight into model assumptions, reasoning processes, and potential consequences of different actions. This shared engagement supports trust, aligns expectations, and enables more nuanced and personalized care decisions.

Finally, PDTs emphasize bidirectional influence between human input and computational models. Patient and caregiver feedback, such as corrections, contextual annotations, or deviations from predicted behavior, can refine how outputs are interpreted and how future interactions are configured. However, such feedback should not be treated as a uniform or sufficient mechanism for correcting bias across all users. Its value depends on whether participation is meaningful, attributable, and adequately supported; when those conditions are absent, PDTs still require clinician oversight and other governance safeguards to prevent model outputs from acquiring unwarranted authority.

Together, these characteristics distinguish PDTs not only from conventional expert-oriented systems but also from approaches in which participation is concentrated primarily at the stage of system design. By centering epistemic agency, boundary object functionality, and interactive sensemaking, PDTs provide a conceptual foundation for reimagining how AI-enabled systems can support shared understanding and democratic engagement in chronic care.

The feasibility and value of PDTs are likely to vary across chronic care contexts. PDTs are most plausibly suited to conditions in which care depends on ongoing self-management; recurrent behavioral trade-offs; and sufficient opportunity for reflection, communication, and iterative dialogue, such as type 2 diabetes, hypertension, and selected forms of cardiovascular risk management. Their applicability is less straightforward in contexts where direct participation is developmentally, cognitively, or structurally constrained. In pediatric chronic care, for example, participation is often necessarily mediated through parents or guardians; in advanced dementia or other conditions involving substantially impaired decisional capacity, sustained interpretive dialogue may be limited or infeasible. Similarly, patients with low digital literacy, limited language concordance, or unreliable access to digital tools may be excluded from participatory features unless accommodations, supported pathways, and nondigital alternatives are deliberately built in. In such settings, participation may still occur in mediated form, but it should not be treated as equivalent to first-person patient engagement. Therefore, PDTs should be understood not as a universal model for all chronic illnesses but as a condition-sensitive design framework whose appropriateness depends on the clinical scenario, the capacities of the people involved, and the organizational and relational supports available to make participation meaningful in practice.

In the next section, we translate this definition into a conceptual framework that illustrates where and how participation can be operationalized within digital twin architectures.

## A Conceptual Framework for PDTs

To operationalize the concept of PDTs, we propose a layered conceptual framework—the PDT framework—that makes explicit where and how participation occurs within digital twin–enabled chronic care systems. This framework reframes digital twins not as isolated technical artifacts but as sociotechnical infrastructures that integrate computation, interaction, and governance to support shared understanding and collaborative decision-making.

### Core Data and Modeling Layer

At the core of the framework is the data and modeling layer, which corresponds to the traditional technical foundation of a digital twin. This layer integrates longitudinal clinical data; patient-generated data (eg, wearable sensors and self-reports); and computational models that support state estimation, simulation, and forecasting. Its primary function is to represent the evolving physiological dynamics relevant to chronic care.

In conventional digital twin architectures, this layer is largely invisible to patients and is accessed primarily by clinicians or technical experts. In participatory frameworks, while the computational complexity may remain hidden, the provenance and selection of data must be transparent, allowing patients to verify what information is driving the model. Insight into data sources such as recent activity, sensor readings, or contextual annotations enables patients to recognize omissions, question assumptions, and meaningfully engage with model outputs without requiring technical expertise.

### Interaction and Translation Layer: The Role of GenAI

Surrounding the core technical layer is the interaction and translation layer, where participation becomes possible. This layer mediates between mathematically complex models and human understanding, enabling users to engage with digital twin outputs through dialogue, exploration, and explanation.

GenAI, particularly LLMs, can play an important but bounded role at this layer. Rather than functioning as autonomous decision-makers, LLMs are better understood as translational agents that help render digital twin outputs more accessible through plain-language explanation, interactive questioning, and scenario-based reasoning. Used in this constrained way, GenAI can support “why” and “what-if” inquiries; help elicit missing contextual information; and make model-based trade-offs more discussable for patients, caregivers, and clinicians [11].

At the same time, this layer introduces nontrivial risks. LLMs may hallucinate content; generate fluent but incorrect explanations; respond inconsistently across prompts or sessions; and struggle to represent uncertainty faithfully, especially when numerical risk or confidence estimates are implied [11-13]. In a chronic care setting, these failure modes could distort rather than clarify the relationship among model output, lived context, and clinical judgment.

For this reason, GenAI within PDTs should be constrained by design. It should be grounded in traceable digital twin data and scenario outputs; limited to interpretable tasks such as explanation, contextual elicitation, and comparison of predefined alternatives; and paired with safeguards such as explicit source attribution, audit trails, uncertainty signaling, and clinician review in higher-risk situations [14,15]. Under these conditions, the interaction layer can support shared sensemaking without treating conversational fluency itself as evidence of clinical reliability.

### Governance and Social Layer

The outer layer of the framework is the governance and social layer, which situates PDTs within broader ethical, organizational, and relational contexts. Participation in chronic care is not only a matter of interface design; it also depends on how responsibility, authority, and accountability are negotiated among patients, clinicians, caregivers, and health systems.

This layer encompasses mechanisms for communicating uncertainty, managing consent, providing avenues for recourse when models fail, and supporting shared responsibility for outcomes. By explicitly addressing what happens when a digital twin’s guidance is questioned or ignored or leads to adverse outcomes, participatory systems acknowledge the inherently probabilistic nature of model-driven care. This governance layer is also where issues of equity, trust, and democratic deliberation become salient, ensuring that participation is accompanied by meaningful safeguards rather than symbolic inclusion.

### Locating Participation Across Layers

A key contribution of this framework is its explicit identification of where participation occurs. Participation is not limited to data entry at the technical core but emerges primarily at the interaction and governance layers, where patients can (1) interpret and question model outputs, (2) verify and annotate data inputs, (3) explore alternative scenarios and trade-offs, (4) participate in shared decision-making conversations, and (5) exercise recourse when recommendations conflict with lived experience.

**Figure 1.**
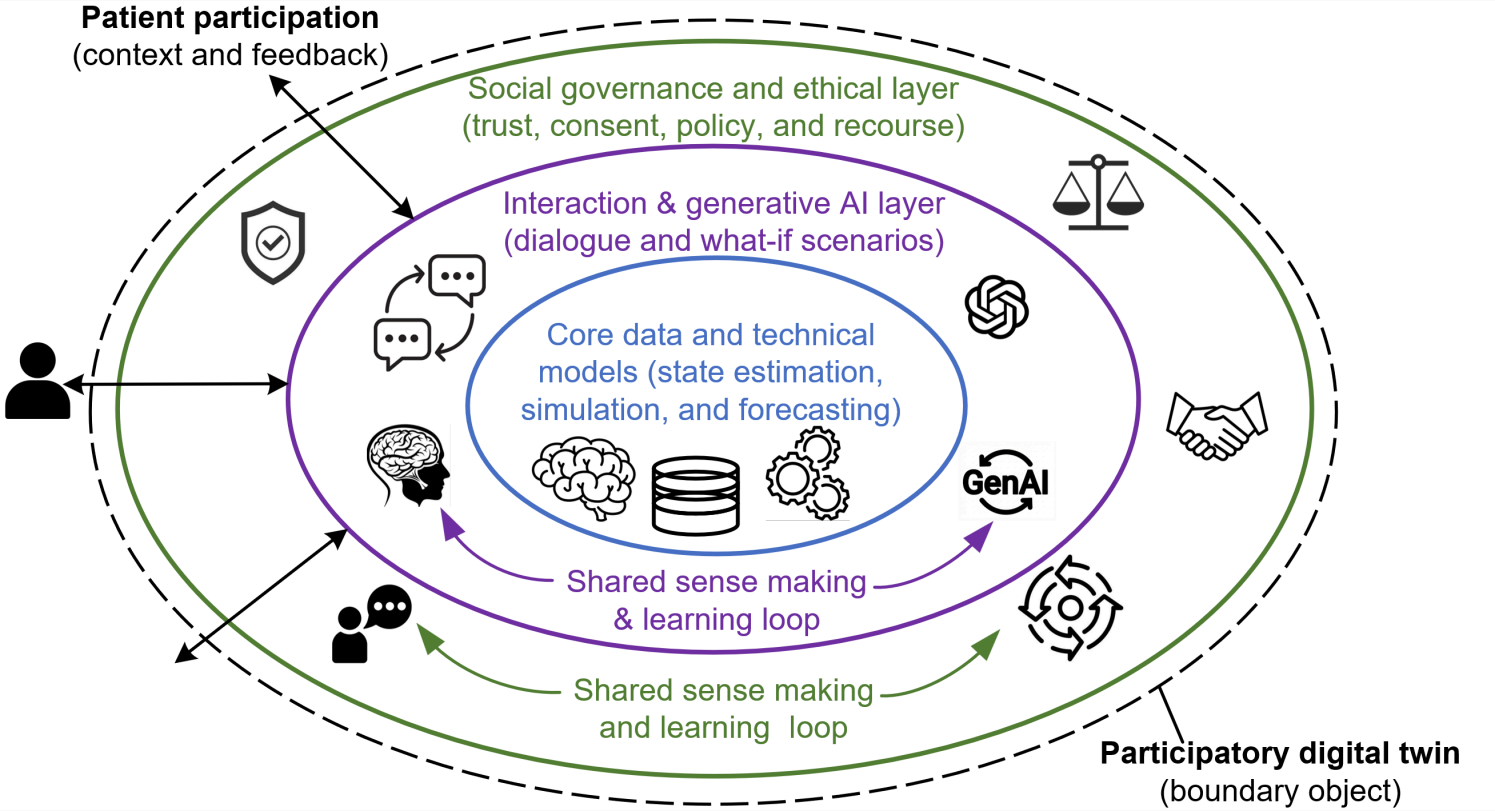
The layered participatory digital twin conceptual framework depicting participatory digital twins as boundary objects that connect computational models, generative artificial intelligence (AI)–mediated interaction, and social governance through a shared representation that can be interpreted differently yet used coordinately by patients, caregivers, clinicians, and health systems. By visualizing participation as a structural feature rather than an afterthought, the framework highlights how digital twins can evolve from expert-oriented tools into shared systems that support understanding, agency, and trust in chronic care.

Through feedback loops that connect these layers, patient input can inform interpretation and guide ongoing system refinement, helping ensure that digital twins remain aligned with patients’ changing realities over time. This layered structure is summarized in Figure 1.

Table 1 summarizes the conceptual shift from expert-oriented digital twins to PDTs across key design dimensions, situating the proposed framework within broader paradigmatic differences in how digital twins are designed and evaluated. Textbox 1 illustrates this contrast through a comparative vignette that shows how an expert-oriented digital twin and a PDT may lead to different user experiences and care decisions.

**Table 1.** Contrasting design paradigms for health digital twins.

Design dimension	Expert-oriented digital twins (current state)	Participatory digital twins (proposed state)
Primary goal	Optimization of clinical outcomes and predictive accuracy	Shared understanding, epistemic agency, and trust
Interaction model	Directive: alerts, risk scores, and prescriptive recommendations	Dialogic: exploration of “what-if” scenarios, trade-offs, and alternatives
Role of the user	Data source: passive recipient of model outputs	Knowledge partner: active contributor of context and constraints
Handling of uncertainty	Hidden: uncertainty is minimized or smoothed over to project confidence	Visible: uncertainty is visualized as a feature to support calibration of trust
Feedback loop	System centric: data used primarily to retrain the model (technical loop)	Human centric: user input refines model interpretation and context over time
Success metrics	Low error rates; adherence to recommendations	User comprehension; perceived engagement and agency; shared decision quality; sustained use over time; and trust, acceptability, and implementation fit (Textbox 1)

**Textbox 1. Comparative vignette: from “black box” to “shared sandbox.”**

**Scenario**

- Maria, an administrator aged 55 y with type 2 diabetes, has been experiencing erratic glucose levels due to workplace stress. She uses a digital twin app to manage her insulin.

**Case A: the standard (expert-oriented) digital twin**

- The interaction: the system analyzes her data and issues a single alert—“Predicted High. Increase Bolus by 2 Units.”
- The user experience: Maria feels hungry (a sign of *low* blood sugar) and confused. She suspects that the artificial intelligence (AI) does not know that she just climbed 3 flights of stairs. Because the reasoning is opaque (“black box”), she distrusts the prediction.
- The outcome: she ignores the alert. Later, her glucose spikes because the AI correctly predicted a delayed rise from protein but failed to explain it. Maria views the tool as unreliable and disengages.

**Case B: the participatory (human-centered) digital twin**

- The interaction: the system detects a trend but opens a “what-if” dialogue. It asks, “I see a trend suggesting a spike. Are you planning any activity?” Maria indicates that she recently took the stairs.
- The translation: the twin updates the graph in real time. It explains, “The stairs help, but the protein from breakfast may still cause a rise in 90 minutes. Here is what happens if you take +2 units vs. if you take a walk.”
- The outcome: Maria understands *why* the spike was predicted. She chooses to take a walk (negotiated care). She feels a sense of *epistemic agency*—she used the tool to make sense of her body rather than just following orders.

## Design Principles for PDTs

### From Framework to Design Principles

Translating PDTs from concept to practice requires a shift in design priorities. Rather than optimizing solely for predictive performance or computational efficiency, PDTs must be designed to support understanding, agency, and trust over time. On the basis of the framework described above, we propose the following design principles to guide the development of PDTs for chronic care.

### Design for Shared Understanding, Not Just Optimization

Traditional digital twins are often optimized to recommend a single “best” action based on model outputs. PDTs, in contrast, should prioritize shared understanding over directive efficiency. Systems should present multiple plausible scenarios, explain trade-offs, and support discussion rather than issuing unilateral recommendations. This shift recognizes that optimal decisions in chronic care are context dependent and value laden, not purely computational.

### Translate Models Into Dialogue

Mathematical models and simulations must be made accessible through interaction. Therefore, PDTs should incorporate translation mechanisms, potentially including GenAI-mediated dialogue, that allow users to ask questions, request clarification, and explore alternatives in plain language. However, the translation layer should be constrained to tasks for which grounding is possible: explaining traceable model outputs, eliciting missing context, comparing predefined scenarios, and signaling uncertainty rather than inventing new clinical rationales or offering ungrounded advice [12-15]. Dialogue in this sense is a means of structuring shared interpretation and supporting health literacy, not of delegating clinical judgment to a conversational model.

### Visualize Uncertainty as a Feature

Uncertainty is inherent in model-driven predictions, particularly in chronic care contexts characterized by incomplete data and behavioral variability. Rather than obscuring uncertainty, PDTs should make uncertainty visible through intuitive visualizations and explanations. Communicating what the system “knows,” “infers,” and “guesses” allows patients and clinicians to calibrate trust appropriately and make more informed decisions.

### Enable User Correction and Contextualization

PDTs should allow users to identify missing, outdated, or misinterpreted context and annotate model inputs accordingly. Features that enable patients to indicate stress, unusual activity, dietary changes, transportation barriers, work constraints, caregiving responsibilities, medication affordability, or other relevant social drivers of health transform user input from passive data contribution into meaningful knowledge participation. Such information need not always be modeled as a permanent variable in the core system; in many cases, it may be more appropriately incorporated as time-sensitive contextual annotation that helps explain or reinterpret model outputs in light of lived circumstances. This design helps ensure that model interpretations remain ecologically valid and aligned with everyday chronic care.

### Support Negotiated, Not Directive, Care

In participatory systems, care decisions emerge through negotiation, not compliance. Digital twins should support exploration of trade-offs between competing goals, such as glycemic control, quality of life, and daily constraints, rather than prescribing fixed actions. By framing recommendations as options to be discussed rather than orders to be followed, PDTs foster agency, trust, and long-term engagement.

### Design for Accountability, Recourse, and Trust

Participation without safeguards risks shifting responsibility without authority. Therefore, PDTs must include mechanisms for accountability and recourse that clarify how decisions are documented; how disagreements are handled; and what happens when model-guided recommendations are questioned, overridden, or followed with adverse consequences. Participatory interaction should complement rather than replace clinical judgment. When patients cannot engage directly or consistently because of cognitive, linguistic, or digital constraints, PDTs should rely on clinician-mediated or caregiver-supported pathways with explicit attribution of who contributed which observations, preferences, or annotations rather than treating proxy input as interchangeable with first-person patient knowledge. In addition, conversational and explanatory functions should remain bounded by traceable model outputs and established governance rules so that participatory features expand shared understanding without weakening clinical safeguards or obscuring responsibility.

## Implications for Participatory Medicine

Reframing digital twins as participatory systems has important implications for participatory medicine, particularly in the context of chronic care, where sustained engagement, shared understanding, and trust are central to effective management. PDTs extend the principles of participatory medicine beyond information access, positioning AI-enabled systems as active mediators of collaboration among patients, caregivers, and clinicians.

### Implications for Patients and Caregivers

For patients and caregivers, PDTs offer opportunities to move from passive receipt of recommendations toward meaningful engagement with their own health trajectories. By enabling plain-language explanations, interactive exploration, and insight into uncertainty, participatory systems can support health literacy and foster epistemic agency. For caregivers, this may include structured ways to contribute observations, clarify day-to-day constraints, and participate in shared interpretation when caregiving responsibilities shape how chronic care is managed in practice. PDTs could further support caregiver involvement through role-specific functions such as caregiver-attributed contextual annotations; shared visit preparation summaries; and scenario discussions tied to medication management, daily routines, or other caregiving tasks. These functions are especially important when participation is mediated or shared because caregivers may need support not only in contributing information but also in understanding uncertainty, preserving attribution, and avoiding substitution of caregiver perspectives for first-person patient priorities. This shift is especially relevant for individuals managing chronic conditions in complex social contexts, where decisions involve trade-offs between medical guidance, daily routines, and quality of life. When patients understand why certain outcomes are predicted and how their actions influence those outcomes, digital twins can function as tools for learning and reflection rather than sources of confusion or contributors to the burden of treatment that already characterizes chronic care.

### Implications for Clinicians

For clinicians, PDTs have the potential to support shared decision-making while addressing practical concerns related to time and cognitive load. Although participatory systems introduce an additional layer of interaction, they may ultimately reduce the interpretive burden on clinicians and explanation time by enabling patients to arrive at clinical encounters with a baseline understanding of their health trajectories and the rationale behind potential options. By serving as boundary objects, PDTs can anchor conversations in shared visualizations and scenario-based reasoning, allowing clinicians to focus on judgment, contextualization, and care planning rather than repeatedly translating abstract model outputs. In this way, participatory designs may help mitigate aspects of clinician burnout associated with repetitive explanation and misaligned expectations.

### Implications for Evaluation and Research Practice

The shift toward PDTs also challenges prevailing evaluation practices in health AI research. Traditional metrics focused on predictive accuracy or error reduction are insufficient to capture participatory value [29]. Evaluation should instead examine whether users can meaningfully understand and work with model-driven information, whether the system supports participatory quality during use, and whether these benefits are sustained over time. In addition to comprehension-focused tasks and scenario interpretation, existing instruments from related areas of patient engagement and shared decision-making research can provide a practical starting point, including the Patient Health Engagement scale for engagement orientation [26] and CollaboRATE for patient-reported shared decision-making quality [31]. For a viewpoint-led translational pathway, early studies need not begin with large clinical trials; a feasible progression would be vignette- or simulation-based studies followed by mixed methods pilot studies and short longitudinal deployments to assess whether patients, caregivers, and clinicians continue to use the system to interpret outputs, question assumptions, and contextualize recommendations over time [28]. For GenAI-mediated components, the QUEST human evaluation framework, which covers quality of information, understanding and reasoning, expression style and persona, safety and harm, and trust and confidence, is useful for evaluating dimensions such as information quality, reasoning, safety, and trust [14]. At the broader system level, the AI for IMPACTS framework helps extend assessment beyond model performance to issues such as integration, acceptability, transparency, governance, and real-world scalability [32].

### Implications for Equity, Ethics, and Governance

PDTs raise critical questions about equity, ethics, and democratic governance in AI-enabled care. Systems that prioritize plain-language interaction, transparency, and recourse may improve access to understanding, particularly for populations with lower health literacy or limited clinical support [12,33]. At the same time, equitable participation cannot be assumed. Contextual factors and social drivers of health may enter PDTs through patient-reported or caregiver-attributed annotations, structured prompts, visit preparation summaries, or clinician-documented contextual notes tied to specific scenarios or periods of care rather than being indiscriminately absorbed as permanent model variables. Because such information may be financially, relationally, or logistically sensitive, PDTs should support selective disclosure; distinguish between information stored longitudinally and information used transiently for interpretation; and make explicit who contributed, can view, can modify, and can act on that information. In contexts where participation is mediated, proxy input should be treated as supportive and explicitly attributed, not as an automatic substitute for first-person patient priorities.

Participatory feedback may help surface missing context, mistaken assumptions, or forms of misalignment that models might otherwise overlook [34]. However, this should be understood as a conditional safeguard rather than as a general mechanism for reducing algorithmic bias. Where meaningful engagement is constrained or unevenly distributed, PDTs should rely on additional protections, including clinician review, documented recourse pathways, role-appropriate proxy involvement, and alternative nondigital routes for communication and decision support. Therefore, responsibility for outcomes should remain explicitly negotiated rather than implicitly shifted onto patients or caregivers, especially in cases where the capacity to question, contextualize, or challenge model outputs is limited. Under these conditions, participatory design can strengthen transparency and accountability without overstating the universality of participation or the corrective power of feedback alone.

### Broader Sociotechnical Implications

Together, these implications underscore that PDTs are not merely technical enhancements but sociotechnical interventions that reshape relationships, responsibilities, and power in chronic care. By aligning AI design with the values of participatory medicine, digital twins can support more equitable, transparent, and democratically governed forms of care.

## Conclusions

Digital twins are increasingly positioned as foundational technologies for chronic care, yet their full potential will remain unrealized if they are designed solely as expert-oriented systems optimized for technical performance. In this viewpoint, we have argued for a reframing of digital twins as participatory systems that support shared understanding, epistemic agency, and collaborative decision-making among patients, caregivers, and clinicians. By emphasizing interaction, transparency, and governance alongside modeling and simulation, PDTs align AI-enabled care with the lived realities of chronic disease management.

This reframing is both timely and necessary. Advances in GenAI now make it possible to translate complex models into dialogue, while growing attention to health equity, trust, and democratic governance highlights the limitations of opaque, directive AI systems. PDTs offer a pathway to move beyond prediction and toward sensemaking, enabling patients to engage meaningfully with their health data and clinicians to anchor care decisions in shared representations rather than unilateral recommendations.

Ultimately, PDTs are not simply a new class of tools but a design direction for chronic care contexts in which patients, or appropriately supported care partners, can meaningfully engage with model outputs, uncertainty, and trade-offs over time. By designing digital twins that users can understand, question, and help shape within these contexts, the field can move toward more equitable, transparent, and sustainable models of AI-supported care.

## Abbreviations

AI: artificial intelligence
GenAI: generative artificial intelligence
LLM: large language model
PDT: participatory digital twin
